# Exploring the comorbidity mechanisms between atherosclerosis and hashimoto’s thyroiditis based on microarray and single-cell sequencing analysis

**DOI:** 10.1038/s41598-025-85112-0

**Published:** 2025-01-13

**Authors:** Yirong Ma, Shuguang Wu, Junyu Lai, Qiang Wan, Jingxuan Hu, Yanhong Liu, Ziyi Zhou, Jianguang Wu

**Affiliations:** 1 Department of Postgraduate, Jiangxi University of Chinese Medicine, Nanchang, China; 2 Cardiology Department, Affiliated Hospital of Jiangxi University of Chinese Medicine, Nanchang, China; 3 Neurology Department, Jiangxi Province Hospital of Integrated Chinese & Western Medicine, Nanchang, China

**Keywords:** Atherosclerosis, Hashimoto’s thyroiditis, Bioinformatics, Immune infiltration analysis, Microarray analysis, Single-cell sequencing analysis, Autoimmunity, Biomarkers, Cardiology

## Abstract

Atherosclerosis (AS) is a chronic vascular disease characterized by inflammation of the arterial wall and the formation of cholesterol plaques. Hashimoto’s thyroiditis (HT) is an autoimmune disorder marked by chronic inflammation and destruction of thyroid tissue. Although previous studies have identified common risk factors between AS and HT, the specific etiology and pathogenic mechanisms underlying these associations remain unclear. We obtained relevant datasets for AS and HT from the Gene Expression Omnibus (GEO). By employing the Limma package, we pinpointed common differentially expressed genes (DEGs) and discerned co-expression modules linked to AS and HT via Weighted Gene Co-expression Network Analysis (WGCNA). We elucidated gene functions and regulatory networks across various biological scenarios through enrichment and pathway analysis using Gene Ontology (GO) and Kyoto Encyclopedia of Genes and Genomes (KEGG). Core genes were identified using Cytoscape software and further validated with external datasets. We also conducted immune infiltration analysis on these core genes utilizing the CIBERSORT method. Lastly, Single-cell analysis was instrumental in uncovering common diagnostic markers. Based on differential analysis and WGCNA, we identified 119 candidate genes within the cohorts for AS and HT. KEGG and GO enrichment analyses indicate that these genes are significantly involved in antigen processing and presentation, along with various immune-inflammatory pathways. Two pivotal genes, PTPRC and TYROBP, were identified using five algorithms from the cytoHubba plugin. Validation through external datasets confirmed their substantial diagnostic value for AS and HT. Moreover, the results of Gene Set Enrichment Analysis (GSEA) indicated that these core genes are significantly enriched in various receptor interactions and signaling pathways. Immune infiltration analysis revealed a strong association of lymphocytes and macrophages with the pathogenesis of AS and HT. Single-cell analysis demonstrated predominant expression of the core genes in macrophages, monocytes, T cells and Common Myeloid Progenitor (CMP). This study proposes that an aberrant immune response might represent a shared pathogenic mechanism in AS and HT. The genes PTPRC and TYROBP are identified as critical potential biomarkers and therapeutic targets for these comorbid conditions. Furthermore, the core genes and their interactions with immune cells could serve as promising targets for future diagnostic and therapeutic strategies.

## Introduction

Atherosclerosis (AS) is a chronic inflammatory disease marked by endothelial cell damage and cholesterol accumulation, which initiates an immune-inflammatory response, resulting in vessel wall thickening and plaque formation^1^. This disease process is a primary contributor to cardiovascular diseases worldwide, substantially altering vascular structure and function, and is strongly linked with critical complications such as myocardial infarction and stroke^2,3^. Driven by shifts in global lifestyles and the rise in aging populations, the incidence of AS continues to grow each year. The research delineated in the Global Burden of Disease Study highlights a significant rise in the incidence of cardiovascular diseases over a span from 1990 to 2019, where the number of cases escalated from 271 million to an alarming 523 million. Additionally, deaths attributed to cardiovascular conditions showed a consistent upward trajectory, climbing from 12.1 million to 18.6 million^4,5^. On a different note, Hashimoto’s Thyroiditis (HT), a prevalent autoimmune disorder of the thyroid, is marked by persistent thyroid gland inflammation and the generation of autoantibodies that frequently result in hypothyroidism. The disease typically presents with symptoms of hypothyroidism and thyroid enlargement, and in severe cases, it may precipitate a temporary hyperthyroid phase^6^. The pathogenesis of HT is multifaceted, involving genetic, environmental, and immunological factors^7,8^. Furthermore, HT often manifests alongside other autoimmune disorders, including rheumatoid arthritis and systemic lupus erythematosus, indicating a more extensive pattern of immune system malfunction^9^. Patients with HT exhibit an elevated cardiovascular (CV) risk. Numerous studies have demonstrated that hypothyroidism heightens the risk of cardiovascular diseases and AS, exhibiting strong independent correlations with aortic AS and myocardial infarction^10,11^. Moreover, a meta-analysis by Ochs et al. revealed that the risk of coronary heart disease (CHD) in individuals with subclinical hypothyroidism increases by approximately 20%. These findings highlight the tight link between thyroid dysfunction and AS. The thyroid gland plays an essential role in regulating metabolism and cardiovascular function. Its dysfunction can directly impact vascular health and exacerbate the progression of AS via effects on lipid metabolism, endothelial function, and inflammatory responses^10,11^. Therefore, delineating the interrelationship between HT and AS is essential for the effective prevention and management of cardiovascular diseases.

Although the precise mechanisms linking AS and HT remain elusive, both conditions underscore the critical role of inflammation and immune system dysregulation. AS typically begins with the subendothelial accumulation of low-density lipoprotein (LDL), provoking a localized inflammatory response^12^. This inflammation is intensified as macrophages ingest LDL and transform into foam cells, leading to endothelial damage and hardening of the vascular wall^13^. Conversely, the pathogenesis of HT involves intricate interactions among various immune cells and inflammatory mediators within the thyroid gland. Notably, there is a marked increase in T lymphocytes, B lymphocytes, and their subsets, which exacerbate the condition by secreting a range of cytokines (such as IL-1, IL-6, TNF-α) and chemokines (such as CXCL10, CCL2). These immune mediators not only sustain the chronic inflammatory state in the thyroid but also induce structural and functional impairments^6,14^. Furthermore, both diseases share a heightened immune-inflammatory response, exemplified by elevated levels of biomarkers like C-reactive protein (CRP), interleukin-6 (IL-6), and tumor necrosis factor (TNFα), indicating active inflammation and reflecting disease severity and progression^15–18^. This shared characteristic of immune-inflammatory response enhances our understanding of the interaction mechanisms between diseases and supports the development of new therapeutic approaches, thereby potentially improving treatment efficacy. Therefore, comprehensive research into these common pathological features is vital for devising broad-spectrum therapeutic strategies.

**Fig. 1:**
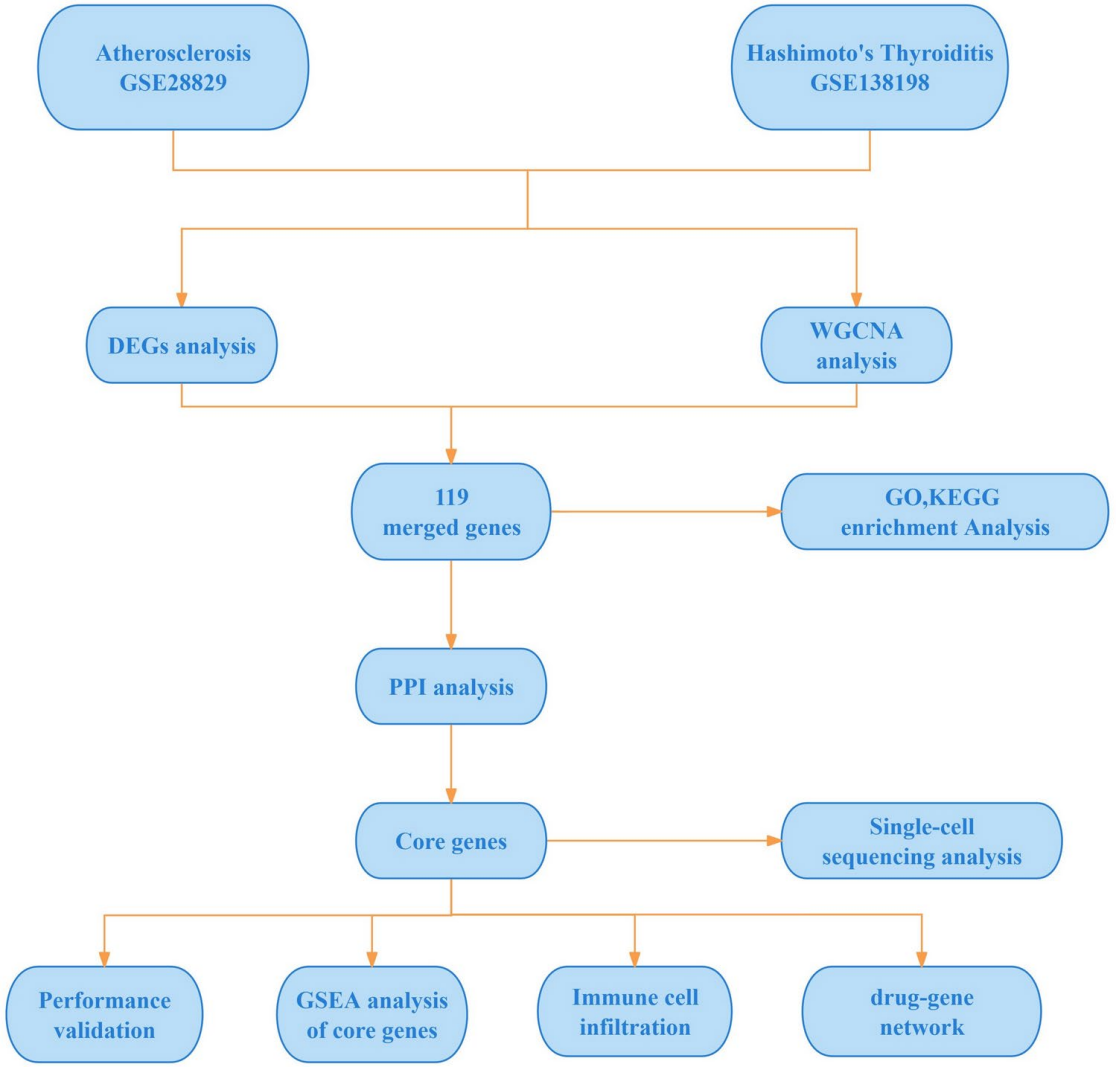
Workflow of the analysis.

Despite numerous studies establishing a correlation between AS and HT, a clear consensus on their common etiology and pathogenesis remains elusive. Consequently, this study aims to enhance targeted clinical interventions by thoroughly analyzing the shared pathogenic mechanisms of these diseases. Leveraging gene expression profiles from public databases, we conducted DEG analysis and WGCNA to identify key genes implicated in both conditions. Additionally, through functional enrichment analysis, we explored relevant biological pathways. Utilizing five algorithms from the Cytohubba plugin, this study also delineated the core genes of these diseases and examined their associations with immune cell infiltration in AS and HT. Employing single-cell technology, we conducted a deeper analysis of these core gene expressions within diverse immune cells from patients with AS. The methodology and principal findings of this research are depicted in Fig. 1. Through this comprehensive series of analyses, we aspire to deepen our understanding of the comorbidity mechanisms between AS and HT and to identify potential therapeutic targets that could enhance patient outcomes.

## Materials and methods

### Data source

We sourced transcriptome datasets (GSE100927, GSE28829, GSE138198, and GSE29315) and a single-cell dataset (GSE155512) from the GEO database. Regarding AS, the dataset GSE100927 (platform: GPL17077) comprised 69 atherosclerotic and 35 healthy arterial samples. GSE28829 (platform: GPL570) included 16 late-stage and 13 early-stage atherosclerotic plaque samples. Additionally, GSE155512 (platform: GPL24676) encompassed 3 atherosclerotic samples. For HT, dataset GSE138198 (platform: GPL6244) contained 13 HT and 3 normal thyroid samples, while GSE29315 (platform: GPL8300) included 6 HT and 8 thyroid hyperplasia samples.

### Differential gene expression screening

We performed gene expression analysis on datasets GSE28829 and GSE138198 for AS and HT, respectively. DEGs were identified using the Limma package in R (Version 4.4.0, released on June 10, 2024), adhering to criteria of Log2|fold change (FC)|> 1 and adjusted *p* value < 0.05. We generated volcano plots and heatmaps for the top 20 ranked DEGs using the ‘ggplot’ package. The ‘VennDiagram’ package was then employed to identify common DEGs between AS and HT.

### Construction and module analysis of WGCNA

A methodology known as WGCNA enhances the investigation of biologically meaningful co-expressed gene modules and probes the connections between gene networks and diseases. In our research, we utilized the ‘WGCNA’ package in R to develop a gene co-expression network, targeting the exploration of associations between genes and phenotypes^19^. Initially, we selected the upper quartile of genes displaying the greatest median absolute deviation (MAD). Subsequently, we computed the Pearson correlation matrix for all gene pairings and crafted a weighted adjacency matrix using average linkage along with weighted correlation coefficients. The adjacency matrix was established by applying a ‘soft’ thresholding power (b), which was then converted into a topological overlap matrix (TOM). For clustering genes with analogous expression patterns, we implemented average linkage hierarchical clustering based on TOM-derived dissimilarity, setting a threshold for the minimum module size at 60. Finally, we examined the similarity among genes within these modules, defined a threshold for cutting the module dendrogram, and amalgamated several modules. The WGCNA analysis enabled us to pinpoint significant modules related to AS and HT, and to create visual representations of characteristic gene networks.

### GO and KEGG enrichment analysis

GO and KEGG analysis facilitate systematic annotation and enrichment of gene functions and biological metabolic pathways, thereby enriching our understanding of the functional aspects contained within biological data^20–23^. We performed these analyses using the ‘clusterProfiler’ package in R, identifying significant pathways at a threshold of *P* < 0.05. The results were visualized using the ‘ggplot2’ package.

### PPI network construction and cluster analysis

We employed the STRING database (http://string-db.org, released on June 12, 2024) to extract gene interactions. The resulting protein–protein interaction (PPI) network was visualized utilizing Cytoscape (version 3.10.1, released on June 12, 2024), with an interaction score threshold exceeding 0.4. Further, we performed cluster analysis through the MCODE algorithm integrated within the Cytoscape plugin^24^. The defined parameters for this analysis were a degree cutoff of 2, a node score cutoff of 0.2, a K-core of 2, and a maximum depth of 100.

### Selection and validation of core genes

We utilized five algorithms from the cytoHubba plugin—MCC, MNC, Degree, EPC, and Bottleneck—to identify the top 10 genes ranked by each method. Subsequently, we employed the Jvenn online tool (https://jvenn.toulouse.inrae.fr/app/example.html, released on June 12, 2024)^25^ to intersect the genes identified by these algorithms, identifying two shared core genes. For validation, datasets GSE100927 and GSE29315 were used for AS and HT, respectively, with the expression of core genes confirmed using the ‘ggpubr’ R package. Additionally, we conducted Receiver Operating Characteristic (ROC) curve analysis using the ‘pROC’ R package, utilizing the Area Under the Curve (AUC) as a measure of reliability.

### Gene set enrichment analysis

Upon pinpointing the central genes, GSEA was utilized for AS and HT employing the ‘clusterProfiler’ package. Patients diagnosed with AS or HT were categorized into groups with high and low gene expression, determined by the median expression levels of the central genes. GSEA was then applied to compute enrichment scores for gene sets, which illuminated differing functional phenotypes. Additionally, GSEA facilitated the comparison of biological pathways between the two expression groups, referencing the c5.go.bp.v7.5.1.entrez.gmt gene set. Gene sets achieving a *p* value < 0.05, a normalized enrichment score (NES) > 1, and a false positive rate (FDR) *q*-value < 0.05 were deemed significantly enriched. Enrichment plots prominently displayed the top five activating and inhibiting pathways for each essential gene in both conditions.

### Immune infiltration analysis

Immune cell infiltration was assessed using the CIBERSORT algorithm to investigate the associations between different immune cell populations and disease conditions. This algorithm deciphers the composition of immune cells from gene expression data. Using the CIBERSORT R package^26^ and the LM22 gene signature from the CIBERSORT website, we quantified 22 types of immune cells. Pearson correlation analysis was then used to elucidate the relationships between various immune cell phenotypes and critical genes, which were visually represented in lollipop plots.

### Single-cell sequencing analysis

Due to the absence of suitable single-cell RNA sequencing (scRNA-seq) datasets for HT, we downloaded only the AS scRNA-seq dataset GSE155512 from the GEO database. We conducted downstream analyses using the Seurat R package (version 4.4.0, released on June 16, 2024)^27^. Initially, we created a Seurat object from the read single-cell expression data and conducted quality control, excluding cells with fewer than 50 expressed genes and those with a mitochondrial gene expression ratio exceeding 5%. The data underwent normalization via the LogNormalize technique, followed by the identification of 1500 highly variable genes through the ‘FindVariableFeatures’ function. Principal Component Analysis (PCA) was then executed, along with cluster analysis using Seurat’s ‘FindClusters’ function. Additionally, t-distributed stochastic neighbor embedding (t-SNE) was utilized for nonlinear dimensionality reduction, facilitating the visualization of the data in t-SNE plots.

## Results

### Identification of differentially expressed genes

**Fig. 2:**
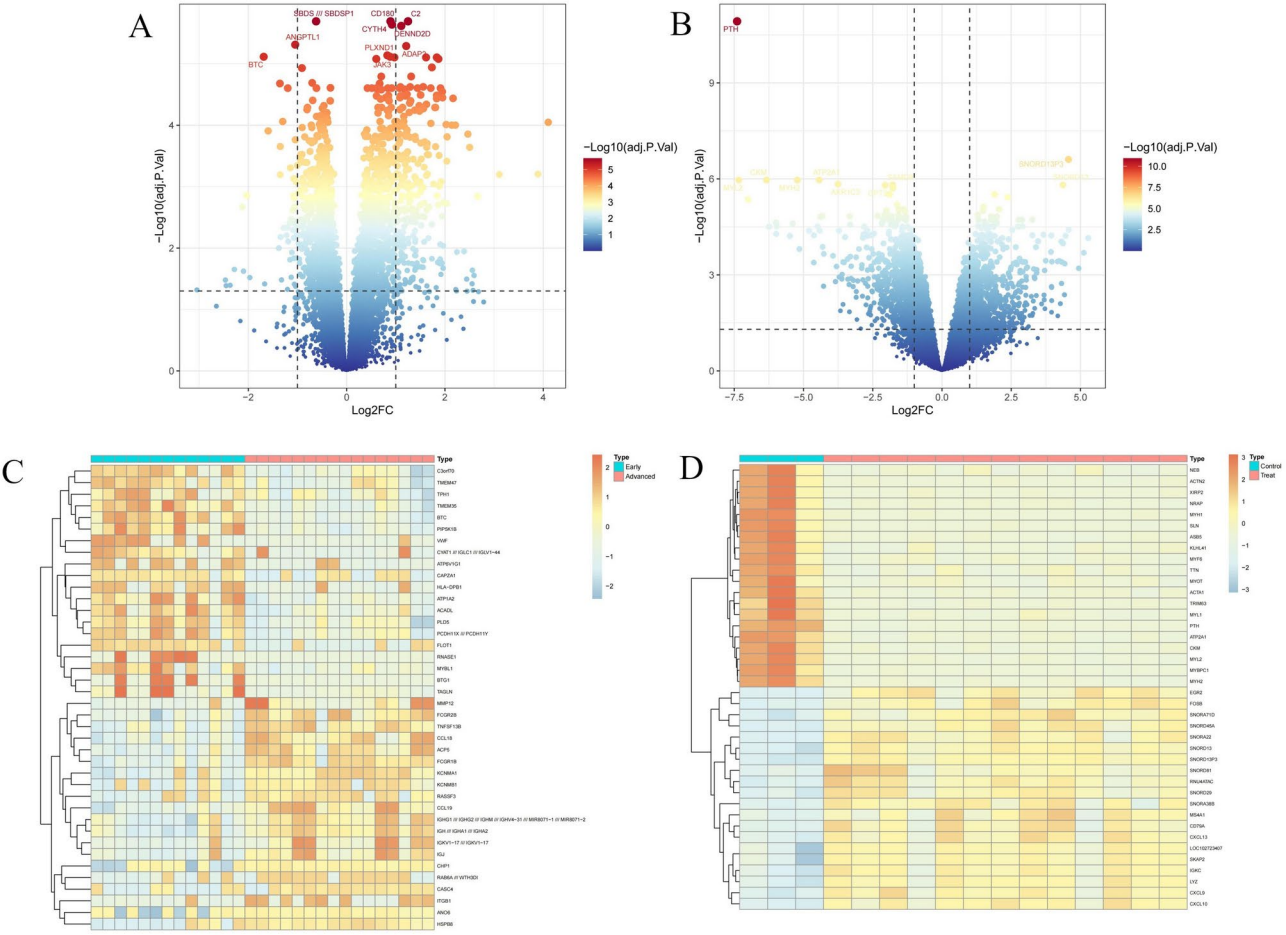
Volcano plot and Heatmap of the DEGs identified from GSE28829 and GSE138198. (**A**,**B**) Volcano map of DEGs fromGSE28829 and GSE138198. (**C**,**D**) Heatmap of DEGs from GSE28829 and GSE138198.

**Fig. 3:**
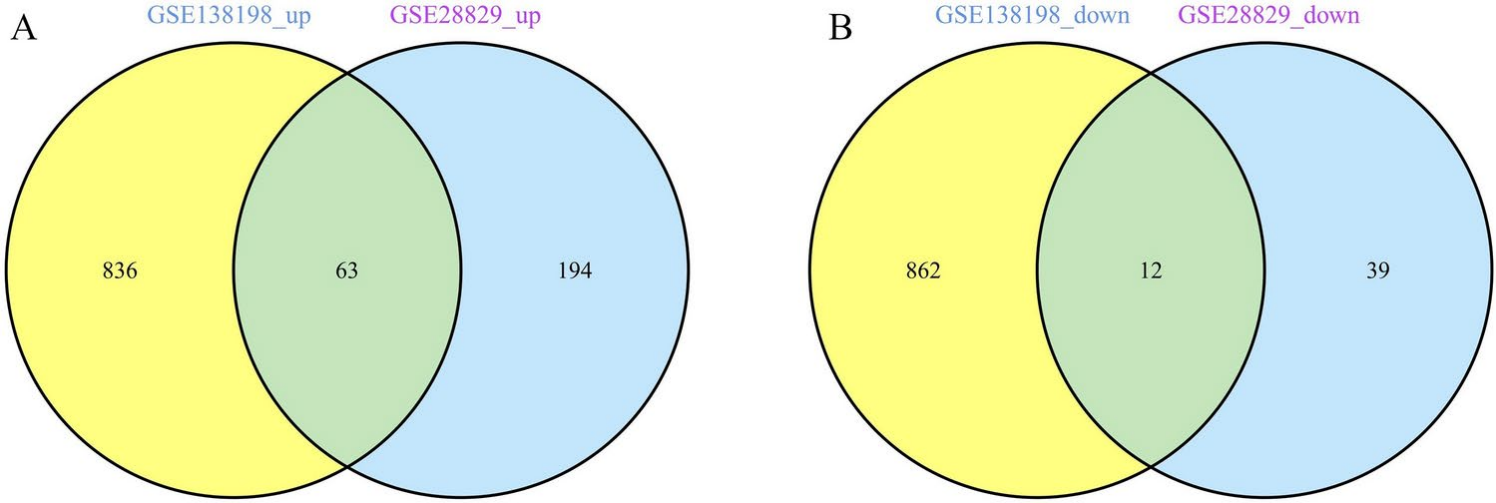
Venn plot of common DEGs between AS and HT. (**A**) Intersection of up-regulated DEGs. (**B**) Intersection of down-regulated DEGs.

In the AS dataset GSE28829, we identified 308 DEGs, of which 257 were upregulated and 51 were downregulated. Similarly, in the HT dataset GSE138198, 1773 DEGs were identified, with 899 upregulated and 874 downregulated. The expression patterns of DEGs in both conditions are depicted using volcano plots (Fig. 2A,B). The top 20 DEGs for AS and HT are represented in heatmaps (Fig. 2C,D). Notably, there is a shared differential expression of 75 genes between AS and HT (Fig. 3A,B).

### WGCNA analysis of AS and HT

**Fig. 4:**
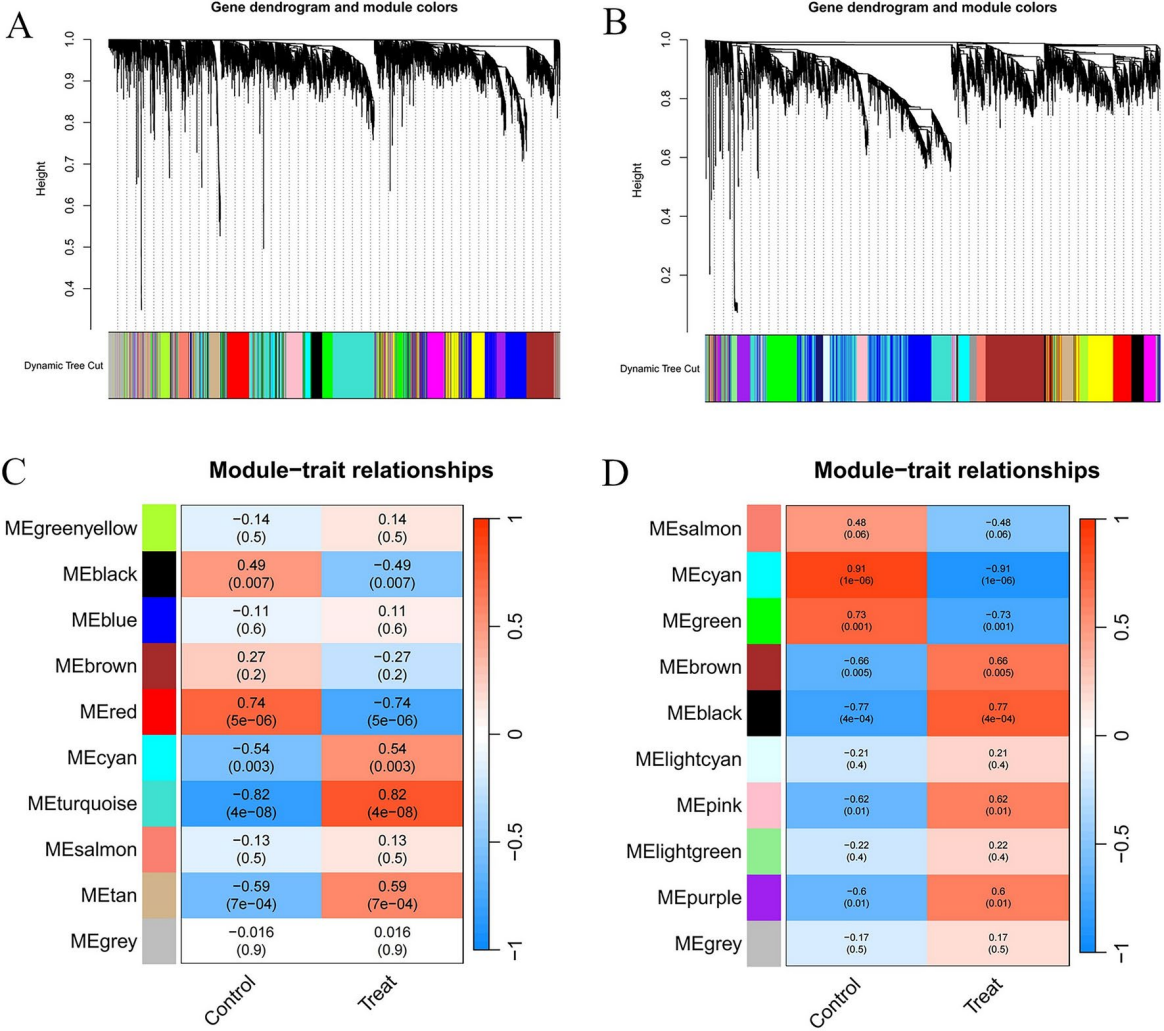
Screening of genes in the GSE28829 and GSE138198 datasets using the WGCNA algorithm. (**A**,**B**) The Cluster dendrogram in GSE28829 and GSE138198. (**C**,**D**) Heatmap illustrating the module-trait relationships in GSE28829 and GSE138198. WGCNA, weighted gene coexpression network analysis.

**Fig. 5:**
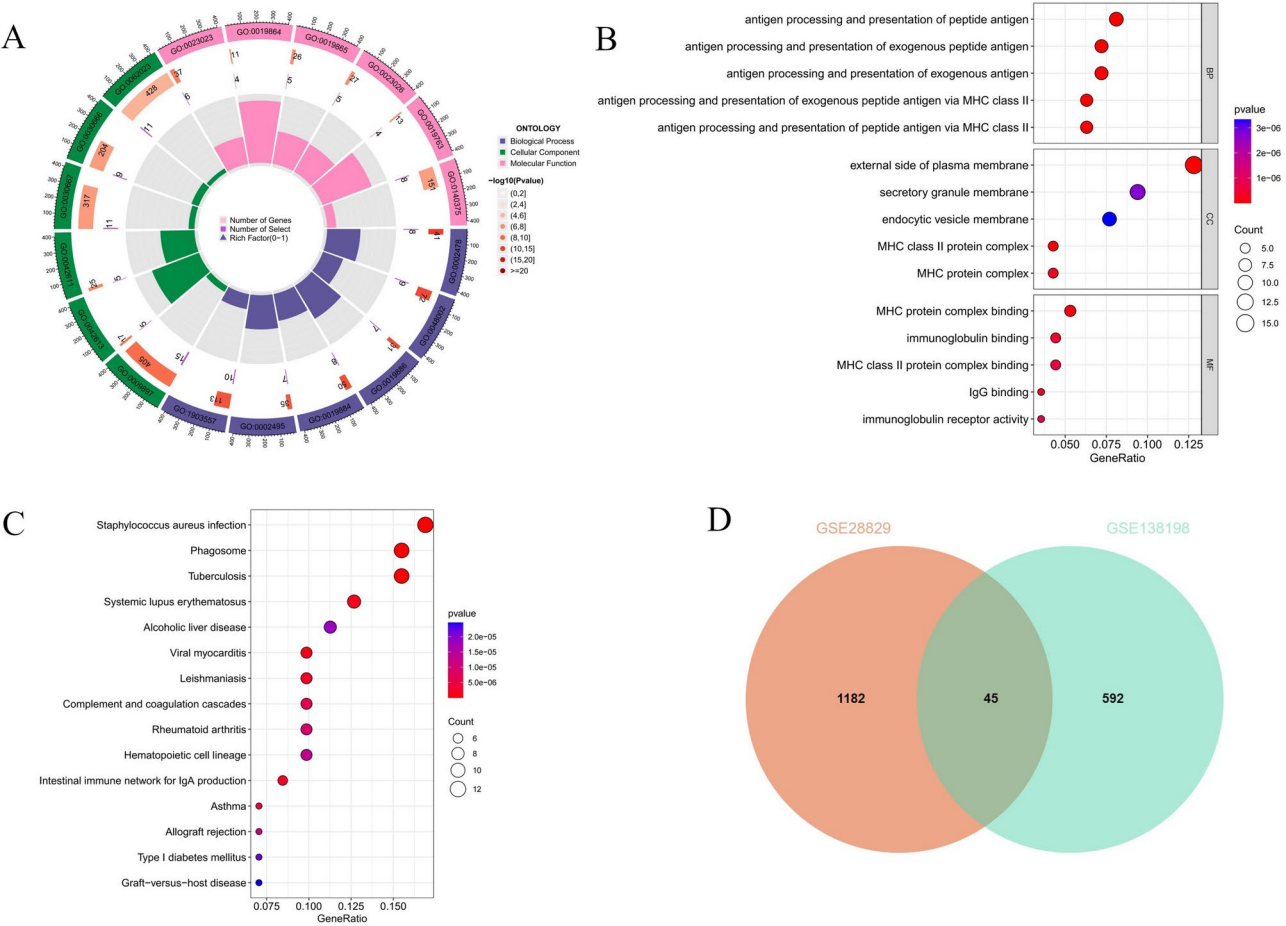
Venn plot of common genes between AS and HT and the PPI network of the merged genes. (**A**) The overlapped genes between the key modules in GSE28829 and GSE138198. (**B**) The PPI network of the merged genes. (**C**) One cluster extracted by MCODE.

We performed WGCNA on the AS dataset GSE28829 and the HT dataset GSE138198 to identify highly associated modules. No significant outliers were found in either dataset. For GSE28829, a “soft” threshold of β = 12 was established based on scale independence and mean connectivity (Supplementary material 1), and 10 modules were detected. Clinical correlation analysis indicated that the “MEturquoise” and “MEred” modules exhibited the strongest positive and negative correlations with AS, respectively (MEturquoise: r = 0.82, *p* = 4e−08; MEred: r = − 0.74, *p* = 5e−06) (Fig. 4A,C). Similarly, for GSE138198, a “soft” threshold of β = 10 was selected, identifying 10 modules. The “MEblack” and “MEcyan” modules showed the highest positive and negative correlations with HT, respectively (MEblack: r = 0.77, *p* = 4e−04; MEcyan: r = − 0.91, *p* = 1e−06) (Fig. 4B,D). Subsequently, these pivotal modules were targeted for further analysis. Intersection analysis of the genes from these key modules identified 45 potential driver genes common to both AS and HT (Fig. 5D).

### Enrichment analysis of genes jointly driving AS and HT

We identified 75 common DEGs between AS and HT and discovered 45 overlapping genes within key modules. To ensure comprehensive coverage of potential key genes, we merged the DEGs with genes from these modules. After eliminating duplicates, 119 candidate genes were identified, which are hypothesized to jointly contribute to the pathogenesis of both diseases (Supplementary material 2). Subsequent GO and KEGG enrichment analyses were conducted on these genes. The GO analysis indicated that biological process (BP) genes are predominantly involved in antigen processing and presentation, crucial for T-cell activation and immune response regulation, through the presentation of exogenous and endogenous peptide antigens via MHC class I and II molecules. Cellular component (CC) genes were mainly enriched in locations such as the external side of the plasma membrane, secretory granule membrane, endocytic vesicle membrane, and MHC protein complex. Molecular function (MF) genes showed enrichment in activities like MHC protein complex binding, immunoglobulin binding, and MHC class II protein complex binding, which are integral to immunoglobulin receptor activity (Fig. 5A,B). The KEGG analysis revealed significant enrichment in pathways associated with Staphylococcus aureus infection, phagosome, tuberculosis, systemic lupus erythematosus, and alcoholic liver disease (Fig. 5C).

### Selection and verification of core genes

**Fig. 6:**
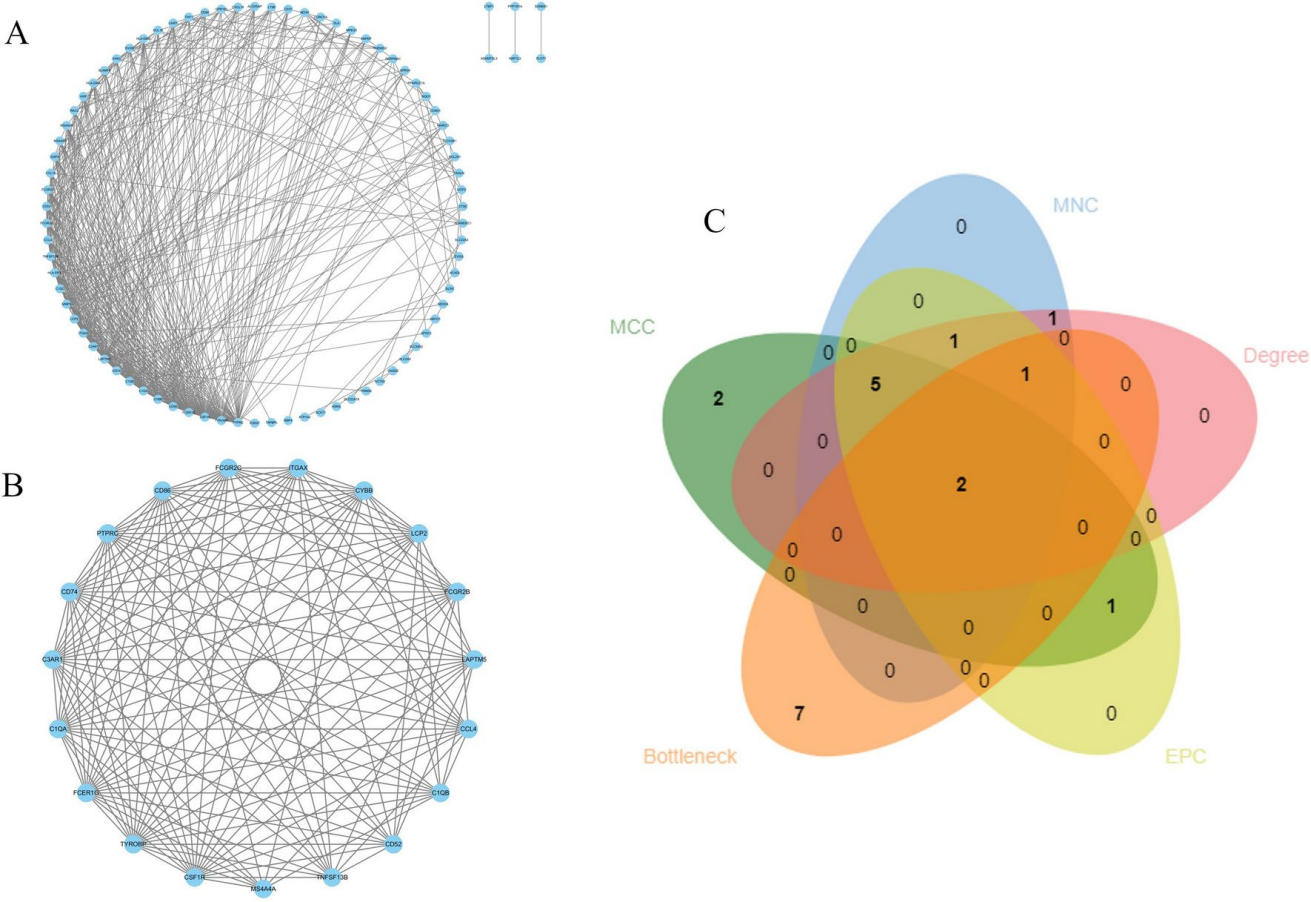
Enrichment analysis of merged genes. (**A**,**B**) Circle plot and bubble plot of GO enrichment analysis includes biological process, cellular component and molecular function. **(C)** Bubble plot of KEGG enrichment analysis. (**D**) Venn diagram of core genes. GO, Gene Ontology; KEGG, Kyoto Encyclopedia of Genes and Genomes.

**Fig. 7:**
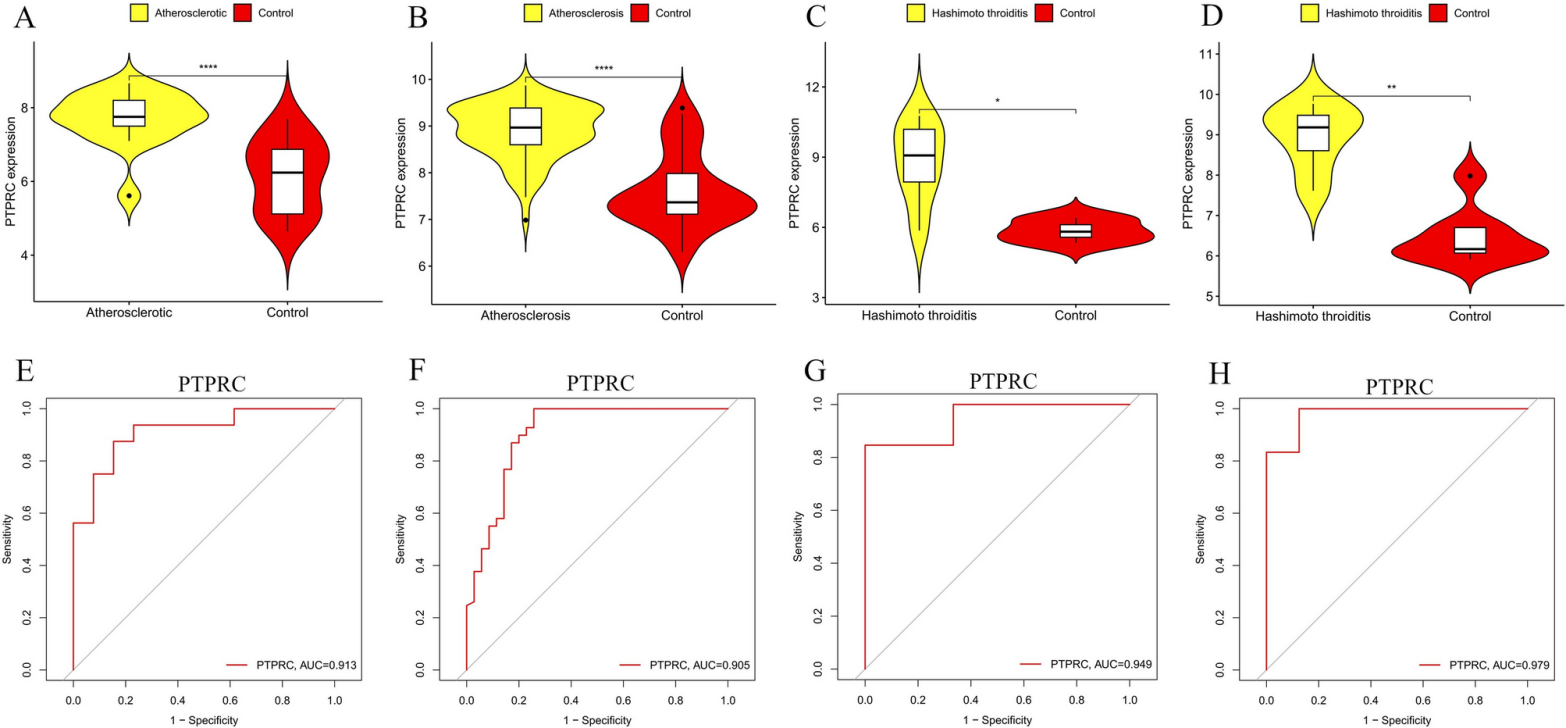
Validation of the expression level and diagnostic efficacy of PTPRC gene. The violin plots of PTPRC gene in GSE28829 (**A**), GSE100927 (**B**), GSE138198 (**C**) and GSE29315 (**D**). The ROC curves of PTPRC gene in GSE28829 (**E**), GSE100927 (**F**), GSE138198 (**G**) and GSE29315 (**H**). *p < 0.05; **p < 0.01;***p < 0.001; ****p < 0.0001.

**Fig. 8:**
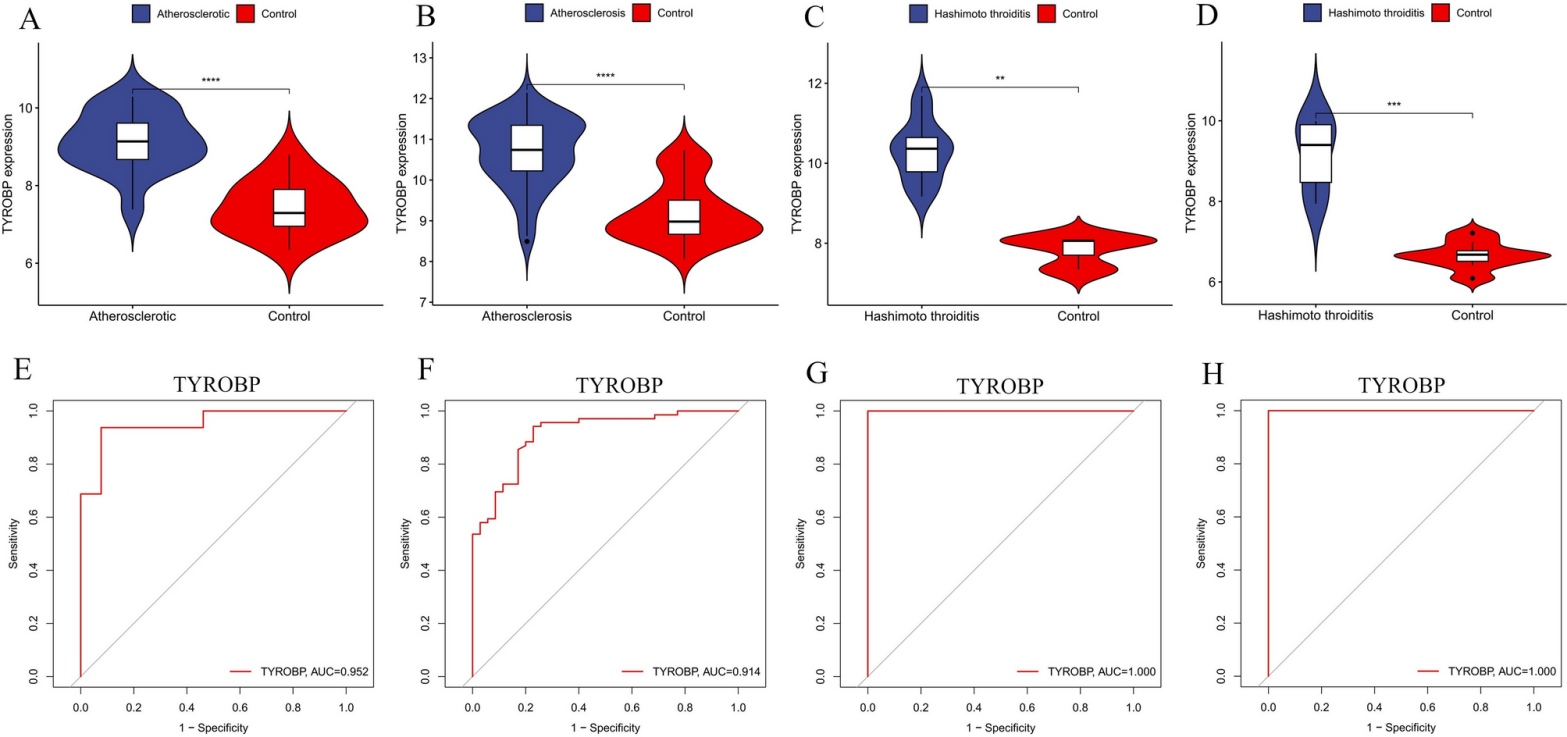
Validation of the expression level and diagnostic efficacy of TYROBP gene. The violin plots of TYROBP gene in GSE28829 (**A**), GSE100927 (**B**), GSE138198 (**C**) and GSE29315 (**D**). The ROC curves of TYROBP gene in GSE28829 (**E**), GSE100927 (**F**), GSE138198 (**G**) and GSE29315 (**H**). *p < 0.05; **p < 0.01;***p < 0.001; ****p < 0.0001.

We inputted 119 candidate genes into the STRING online database and subsequently constructed their PPI network using Cytoscape software, which resulted in 78 nodes and 472 links after isolating genes (Fig. 6A). Using the MCODE plugin, we identified a cluster consisting of 19 nodes and 141 edges, scoring 15.667 (Fig. 6B). We employed five different algorithms to calculate gene scores and identified the top 10 core genes (Fig. 6C). From these analyses, two core genes, PTPRC and TYROBP, emerged (Supplementary material 3). We subsequently validated their expression levels across four datasets, noting that their expression was elevated in AS and HT compared to controls (Figs. 7A–D and 8A–D). Additionally, the diagnostic efficacy of these genes was evaluated across four distinct datasets, with AUC values exceeding 0.9, confirming their high diagnostic potential for AS and HT (Figs. 7E–H and 8E–H).

### GSEA analysis of core genes

**Fig. 9:**
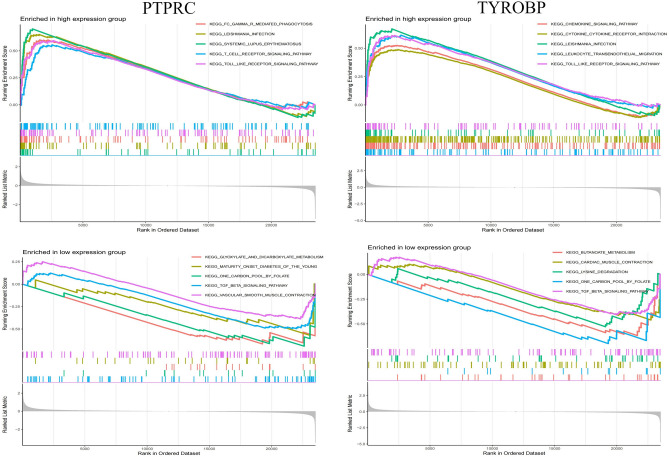
GSEA analysis of core genes (PTPRC and TYROBP) in AS. GSEA Gene set enrichment analysis.

**Fig. 10:**
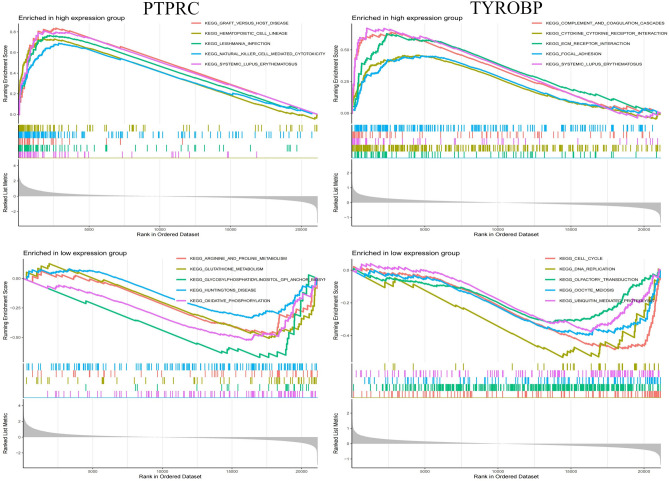
GSEA analysis of core genes (PTPRC and TYROBP) in AS. GSEA Gene set enrichment analysis.

GSEA was performed on two core genes within the AS and HT datasets (Figs. 9, 10). The analysis, conducted using the “GSEA” software package, highlighted the top five upregulated and downregulated pathways. In both disease cohorts, these core genes were implicated in cytokine-cytokine receptor interactions and pathways related to systemic lupus erythematosus. Furthermore, all genes showed enrichment in a variety of immune and inflammatory pathways, including the chemokine signaling pathway, toll-like receptor signaling pathway, T cell receptor signaling pathway, and the complement and coagulation cascades.

### Immune cell infiltration and its association with core genes

**Fig. 11:**
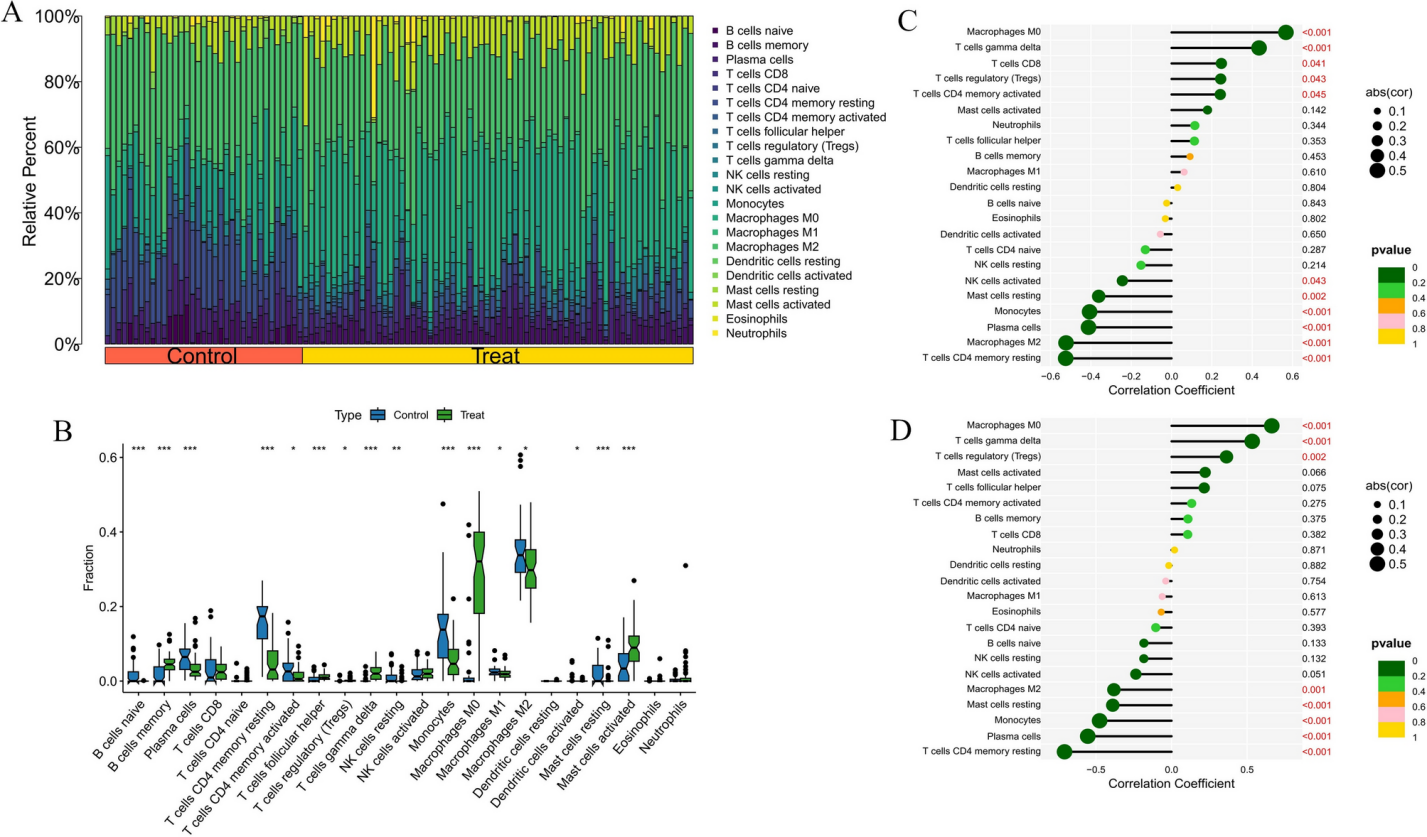
Immune infiltration analysis of AS. (**A**) Histogram of proportion of immune cells. (**B**) Comparison of immune cell proportion between AS and controls (Willcoxon’s test). (**C**) Correlation between PTPRC and immune cells content in AS. (**D**) Correlation between TYROBP and immune cells content in AS. *p < 0.05; **p < 0.01;***p < 0.001.

**Fig. 12:**
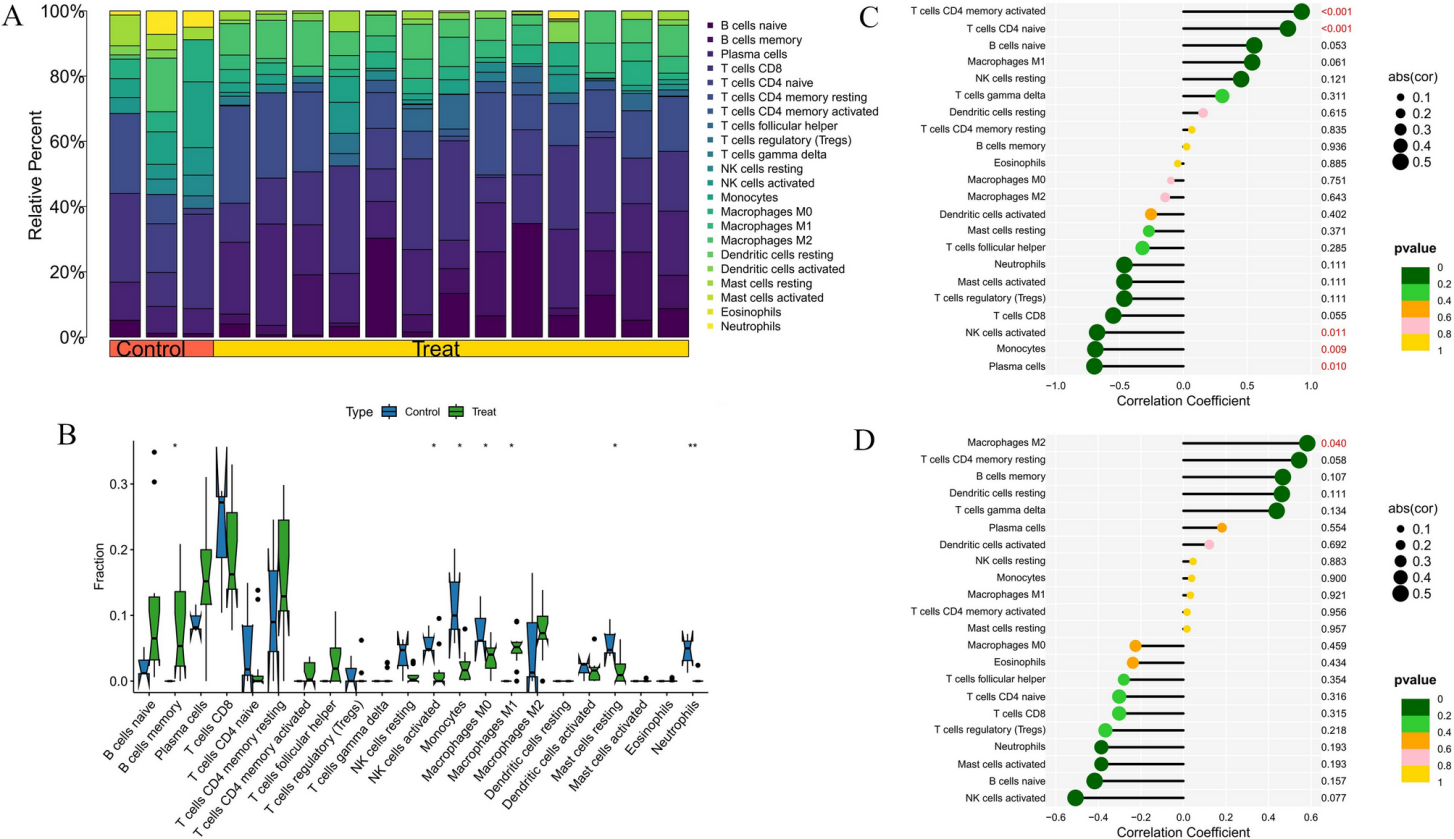
Immune infiltration analysis of HT. (**A**) Histogram of proportion of immune cells. (**B**) Comparison of immune cell proportion between HT and controls (Willcoxon’s test). (**C**) Correlation between TYROBP and immune cells content in HT. (**D**) Correlation between TYROBP and immune cells content in HT. *p < 0.05; **p < 0.01;***p < 0.001.

To elucidate the immune landscape and investigate potential immune mechanisms, we analyzed the distribution of 22 immune cell types in the GSE100927 and GSE138198 datasets using the CIBERSORT algorithm, presenting the results in bar graphs (Figs. 11A and 12A). In the AS cohort, increased counts of memory B cells, regulatory T cells, follicular helper T cells, gamma delta T cells, M0 macrophages, and activated mast cells were observed. Conversely, levels of naive B cells, plasma cells, naive CD4 + T cells, activated CD4 + memory T cells, resting NK cells, monocytes, activated dendritic cells, M1 macrophages, M2 macrophages, and resting mast cells were reduced. In HT, there was an elevation in memory B cells and M1 macrophages, while activated NK cells, monocytes, M0 macrophages, resting mast cells, and neutrophils showed lower levels (Figs. 11B and 12B). In both conditions, memory B cells consistently exhibited elevated levels, while monocytes and resting mast cells showed decreased counts. Additionally, a correlation analysis between core genes and immune cells was conducted (Figs. 11C,D and 12C,D). Spearman correlation tests revealed significant associations between hub genes and macrophages, T cells, and monocytes.

### Expression of core genes in single cells

**Fig. 13:**
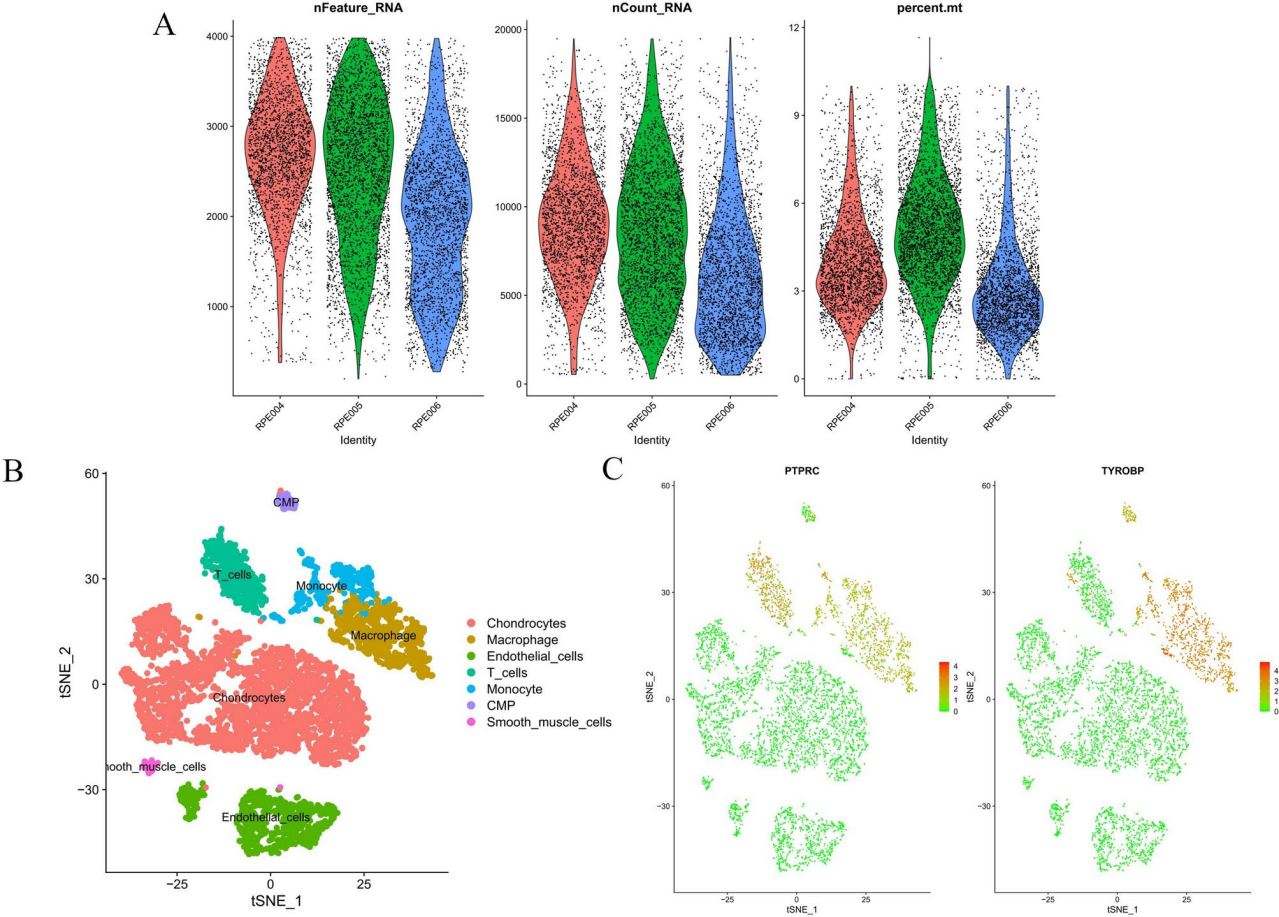
Quality control results for single-cell data are presented as follows: the number of genes, the number of gene reads, and the proportion of mitochondrial genes, listed from top to bottom. (**A**) Post-processing panel. (**B**) Cellular subtypes of AS. (**C**) Scatter plot of the expression of PTPRC and TYROBP.

We acquired single-cell data from GSE155512 and performed single-cell analysis using the Seurat software package, with cell clustering executed via the t-SNE algorithm. Following data quality control, low-quality cells were excluded (Fig. 13A). Cells from three samples were organized into seven subgroups, including chondrocytes, macrophages, endothelial cells, T cells, monocytes, CMP and smooth muscle cells (Fig. 13B). The analysis revealed that PTPRC and TYROBP were predominantly expressed in macrophages, monocytes, T cells and CMP (Fig. 13C).

## Discussion

Research indicates that both AS and HT are associated with immune system involvement and inflammatory responses^28,29^. The development of AS initiates with damage to vascular endothelial cells, which in turn activates the immune system. This activation prominently features inflammatory cells including monocytes, macrophages, and T-cells. These cells migrate to the subendothelial regions of blood vessels, establishing the primary sites for atherosclerotic plaque formation. Notably, macrophages undergo transformation into foam cells through the absorption of oxidized low-density lipoprotein (oxLDL) and the activation of receptors including LOX-1 and CD36, thereby becoming essential components of atherosclerotic plaques. Furthermore, macrophages contribute to the release of inflammatory factors such as IL-1β and TNF-α via the NF-κB pathway, thereby exacerbating the inflammatory response and accelerating the progression of AS^12,13^. Concurrently, T-cells recognize vascular-specific antigens, such as oxidized low-density lipoprotein, and provoke the release of pro-inflammatory cytokines, including IFN-γ, TNF-α, IL-1, and IL-6. This activity intensifies the inflammatory response within plaque regions and promotes the recruitment and activation of additional immune cells^30,31^. Specifically, Th1 cells enhance macrophage uptake and activation of s through their production of IFN-γ. Regulatory T-cells (Tregs), although playing a limited role in controlling these inflammatory responses, contribute to the persistence and exacerbation of the inflammatory milieu. The behavior of these immune cells not only influences the level of inflammation but also potentially affects the stability of the plaques, thereby increasing the risk of rupture—a major risk factor for acute cardiovascular events such as myocardial infarction and stroke^32–34^. In the pathogenesis of HT, specific autoantibodies, including anti-thyroid peroxidase (TPO) and anti-thyroglobulin (Tg) antibodies, play a pivotal role. These antibodies attach to particular target molecules on thyroid cell surfaces, thereby stimulating the immune response. This activation leads to an accumulation of inflammatory cells such as macrophages and T-cells within the thyroid tissue, accompanied by the release of cytokines and chemokines, including IL-1, IL-6, TNF-α and IFN-γ. These mediators not only intensify the inflammatory response and facilitate further migration and activation of immune cells but also directly impair thyroid cell functionality, disrupting their ability to synthesize hormones^35,36^. Moreover, the persistent inflammatory response induces oxidative stress and activates chronic tissue repair mechanisms within the thyroid, which may exacerbate thyroid structure damage. Over time, this chronic damage can result in thyroid tissue fibrosis, a direct outcome of prolonged inflammation^37–39^. In conclusion, macrophages and T-cells play a fundamental role in the pathogenesis of AS and HT, driving disease progression through their involvement in inflammatory processes and immune regulation. Future research is directed towards elucidating the mechanisms of action of these cells, with the aim of developing targeted therapies such as modulating specific receptor activities or enhancing regulatory T-cell functions. These advancements aim to effectively control inflammatory responses, improve treatment outcomes, and pioneer new strategies for disease management. Such progress not only has the potential to enhance patient quality of life but also offers significant theoretical and practical insights for the clinical treatment of cardiovascular and autoimmune diseases.

The precise mechanisms underlying AS and HT remain elusive, which has prompted this study to undertake a comprehensive bioinformatics analysis aimed at elucidating the shared mechanisms, pathways, and immune infiltration characteristics of AS and HT. Our analysis identified two pivotal genes, PTPRC and TYROBP. Results of the GSEA demonstrate a significant association between the elevated expression of specific genes and enhanced interactions with numerous receptors, alongside involvement in various receptor signaling pathways. Further examination of immune infiltration has uncovered a notable correlation between the expression of these genes and the prevalence of macrophages and lymphocytes in the development of AS and HT. Single-cell analysis showed that PTPRC and TYROBP are predominantly expressed in macrophages, monocytes, T cells, and common CMP. Moreover, immune profiles from patients with AS and HT demonstrated increased levels of memory B cells in affected individuals. Consequently, we propose that there may be shared pathogenic processes between AS and HT, possibly linked to memory B cells.

The involvement of B cells in the progression of AS is complex and presents conflicting evidence. Studies have demonstrated that various B cell subtypes exert both promotive and inhibitory effects on atherogenesis. B cells can recognize oxidized low-density lipoprotein (ox-LDL) and generate specific antibodies, leading to the formation of immune complexes. These aggregates build up within the vascular endothelium, possibly triggering inflammatory responses that facilitate the formation and growth of atherosclerotic plaques. Moreover, B cells might secrete pro-inflammatory cytokines, including IL-6 and TNF-α, further exacerbating local inflammation and vascular damage^29,40^. Ox-LDL not only triggers inflammation and oxidative stress in endothelial cells but also facilitates the recruitment and activation of leukocytes by modulating immune cell activity, such as that of macrophages, thereby exacerbating vascular inflammation and dysfunction. These processes underscore the critical role of B cells in AS and highlight their potential as therapeutic targets^12,31,41^. Conversely, regulatory B cells (Bregs), particularly those producing interleukin-10 (IL-10), known as B10 cells, exert substantial anti-inflammatory and immunomodulatory effects in the disease. By secreting IL-10, these B cells help mitigate inflammatory responses, thus safeguarding the vascular wall against further damage. Research indicates that B10 cells’ development and function necessitate a diverse range of antigen receptors and Toll-like receptor (TLR) signaling. These cells are crucial in modulating autoimmunity and inflammation, particularly within the spleens of adult mice, where B10 cells proliferate significantly and are more prevalent in aged mice and those susceptible to autoimmune diseases^42–44^. Furthermore, B cells are crucial in the pathogenesis of HT due to their production of specific autoantibodies, such as TPO antibodies and Tg antibodies. These autoantibodies target and bind to molecules on thyroid cells, triggering immune responses that promote inflammation and the gradual deterioration of thyroid tissue^8,45,46^. Furthermore, studies have shown that B cells significantly influence the pathogenesis of HT by modulating the functions of T cells. During the initial stages of HT, B cells contribute to the regulation of autoimmune responses by secreting immunomodulatory factors, such as IL-10, which significantly impact the behavior of Th1 and Th2 cells and subsequently influence the long-term progression of the disease^47^. Recent advances indicate that targeted therapeutic approaches that focus on B cell functions—such as the use of combined monoclonal antibodies and chimeric molecules of thyroid protein epitopes—may modulate autoreactive B cells and potentially alleviate or reverse the progression of HT^48,49^. This strategy, which involves inhibiting the overactivation of B cells, promoting the apoptosis of specific B cells, and regulating cytokines and chemokines, opens new avenues for treating HT and allows for more precise management of autoimmune thyroiditis. Future studies may delve into methods for modulating the activity or specific functions of B cells to develop novel treatments for both AS and HT. Potential approaches could include small molecule inhibitors, monoclonal antibodies, or other biologics that target B cell surface receptors or secreted cytokines^50^. Additionally, leveraging advanced gene editing technologies, such as CRISPR/Cas9, to precisely regulate specific gene expression in B cells may represent an effective treatment method. The development of these therapeutic strategies necessitates a profound understanding of the role of B cells in these diseases and a comprehensive grasp of the intricate interactions within the immune system.

PTPRC, also known as CD45, is a tyrosine phosphatase receptor ubiquitously present on all nucleated leukocytes, playing an essential role in the activation of T cells and B cells mediated by antigen receptors^51,52^. PTPRC modulates cell signaling by regulating the activity of Janus kinases (JAK) and Src family kinases (SFKs). It mainly acts by dephosphorylating tyrosine kinases within the JAK family, thereby inhibiting their activity. This function is crucial in both the activation and regulation of immune cells^53–55^. Additionally, PTPRC exhibits various isoforms across different immune cells due to its multiple splicing variants, each significantly influencing cell function and activity. For instance, the CD45RA isoform is primarily expressed in naïve T cells that have not yet been activated by antigens, while the CD45RO isoform is predominantly found in memory T cells. This differential expression of isoforms highlights their distinct roles in cell activation and signal transduction^56^. In the study of autoimmune diseases, memory T cells expressing CD45RO exhibit heightened reactivity to specific autoantigens. Furthermore, inflammatory states and infections modulate the expression of these PTPRC isoforms. For instance, during sepsis, the expression of CD45RO on lymphocytes may decrease, while the expression of CD45RA can vary depending on the cell type and inflammatory stimuli^56,57^. PTPRC’s role in the immune system is extensive, encompassing not only adaptive immunity but also a critical function in innate immunity. For example, in mast cells, PTPRC regulates antigen-induced immune responses through Fc receptor-mediated signaling pathways. In dendritic cells, it acts as a key regulator of TLR signaling, which is essential for pathogen recognition and the activation of innate immune responses^52,58^. The regulatory functions of PTPRC are invaluable in studying AS, notably through its indirect control over the composition and activity of inflammatory cells within plaques. This control is primarily exerted through the anti-inflammatory actions of regulatory T cells and B cells, effectively reducing inflammation and preventing the destabilization of plaques^33^. Numerous studies have established a close association between increased expression of PTPRC and the onset and progression of AS^59–62^. This multifunctional regulatory mechanism highlights the importance of PTPRC as a potential therapeutic target for cardiovascular diseases, offering new avenues for future research aimed at combating AS. Additionally, genetic studies on HT have identified specific variations in the PTPRC gene that increase susceptibility to autoimmune diseases, such as Type 1 diabetes and Graves’ disease. PTPRC may significantly influence aberrant autoimmune responses by regulating lymphocyte signal transduction and activation^63,64^. This implies a possible role for PTPRC in HT, although further studies are required to substantiate this hypothesis. Dysfunctional PTPRC may lead to the dysregulation of autoimmune responses, resulting in attacks on thyroid tissue, which underscores a potentially critical role in HT^65,66^. This connection opens new avenues for future research that could clarify the specific pathogenesis of HT and identify potential therapeutic targets. In conclusion, the roles of PTPRC in studies on HT and AS mutually reinforce its broad applications in immune regulation. Therefore, a deeper exploration of the interactions between PTPRC and other immune regulatory molecules could uncover the complex networks involved in pathological states, setting the stage for the development of more comprehensive treatment approaches.

TYROBP (also known as DAP12) is a transmembrane adaptor protein extensively expressed in human immune cells, including natural killer (NK) cells, macrophages, and specific T cell subsets^67,68^. This protein features an Immunoreceptor Tyrosine-based Activation Motif (ITAM) essential for signaling processes. Upon ligand binding to immune cell surface receptors, TYROBP’s ITAM domain is phosphorylated, initiating the activation of key signaling molecules such as the tyrosine kinases Syk and ZAP70. This activation triggers a cascade of intracellular signaling events, including cell activation, proliferation, cytokine release, and cytotoxic functions^69,70^. The phosphorylated ITAM can also recruit and activate additional crucial signaling molecules, such as phosphoinositide 3-kinase (PI3K), small GTPase RAS, and phospholipase C gamma (PLCγ), facilitating various biological responses like transcription, cell proliferation, and cytokine release^71,72^. Studies have demonstrated that TYROBP plays a pivotal role in the progression of AS through its signaling pathways. In macrophages, TYROBP directly affects lipid uptake and processing. The phosphorylation of TYROBP’s ITAM domain, mediated by Src family kinases, occurs upon receptor activation when macrophages engage low-density lipoprotein (LDL) via receptors such as CD36 and SR-A^73,74^. This phosphorylated ITAM then serves as a signaling hub, further recruiting and activating Syk. Syk’s activation initiates signaling cascades involving pathways such as PI3K and PLCγ. PI3K activation leads to the production of PIP3, which in turn activates the Akt signaling pathway, impacting cell survival and proliferation. Simultaneously, PLCγ activation results in the generation of diacylglycerol (DAG) and inositol trisphosphate (IP3), which regulate intracellular calcium signaling and protein kinase C activation^75,76^. This cascade of activated signaling pathways results in macrophages engulfing and accumulating oxidized LDL, thereby transforming into foam cells. This transformation increases intracellular oxidative stress and inflammatory responses, exacerbating AS^77,78^. Furthermore, TYROBP is crucial in autoimmune diseases like HT, influencing immune responses involving macrophages and NK cells. NK cells, which are capable of producing cytokines such as IL-10, regulate immune balance and play various roles in the initiation, progression, and alleviation of autoimmune and autoinflammatory diseases^79,80^. Studies indicate that in the pathogenesis of HT, the influx of macrophages and dendritic cells is directly triggered by inflammatory events. These cells impact thyroid cell growth and function through pathways mediated by interleukins such as IL-1 and IL-6. This process facilitates lymphocyte recognition of self-antigens, leading to considerable generation of autoreactive CD4 + T cells, CD8 + cytotoxic T cells, and immunoglobulin G (IgG) autoantibodies. Additionally, research suggests that the interplay between macrophages, dendritic cells, and T cells amplifies the inflammatory response, culminating in the production of diverse inflammatory cytokines that are vital for the progression of HT^65,81^. Genetic research also reveals that genetic variations associated with TYROBP may increase susceptibility to autoimmune diseases. Specific genetic markers identified in genome-wide association studies (GWAS) related to autoimmunity may enhance disease risk by influencing molecules in immune regulation pathways, such as TYROBP. These genetic variations significantly affect immune cell functions, particularly those of T cells and regulatory T cells, thereby playing a pivotal role in the development of autoimmune diseases. These findings offer new targets for the treatment of autoimmune diseases and enhance our understanding of their pathogenic mechanisms^82^. Therefore, based on prior research, TYROBP is likely to serve as a biomarker for patients with AS and HT.

This study boasts several significant strengths. We implemented a comprehensive and intricate bioinformatics analysis approach to investigate the interactions between AS and HT. By examining the shared molecular mechanisms and pathways of AS and HT, we pinpointed crucial genes and immune infiltration characteristics. These were corroborated through external datasets, thereby enhancing the predictive accuracy. Our findings potentially illuminate the shared mechanisms underpinning AS and HT. Nevertheless, it is important to acknowledge the limitations of this study. Firstly, the data were sourced from the GEO database, which could have inconsistencies in collection and processing methods, potentially affecting the accuracy and reliability of our analyses. Variations in processing methods and technical platforms across laboratories could introduce biases. Additionally, our analysis primarily focused on gene expression changes without directly measuring protein expression and activity. Considering that gene expression levels do not always correlate with protein functions, our findings necessitate further validation at the protein level. Consequently, future research should prioritize multidimensional data integration, enhance quality control, expand sample sizes, and experimentally validate the functions of identified genes and proteins. Such approaches will foster a more comprehensive understanding of the pathogenic mechanisms of AS and HT and identify more precise clinical treatment targets.

## Conclusion

In this research, we pinpointed two key genes, PTPRC and TYROBP, crucial for AS and HT, and elucidated shared regulatory pathways and common immune characteristics. This led to the development of an effective diagnostic model. Further analysis using CIBERSORT revealed a significant correlation between the expression of these core genes and immune cell infiltration. Additionally, single-cell sequencing analysis demonstrated that these genes are primarily expressed in macrophages, monocytes, T cells, and CMPs. Our findings enhance the understanding of the molecular mechanisms underlying both diseases by highlighting key genes and immune regulatory pathways. These achievements not only deepen our knowledge of the pathologies of AS and HT but also set the stage for further clinical research and therapeutic development.

## Supplementary Information

Supplementary Information 1.

Supplementary Information 2.

Supplementary Information 3.

## Data Availability

The datasets GSE100927, GSE28829, GSE155512, GSE138198, and GSE29315 for this study can be found in the https://www.ncbi.nlm.nih.gov/geo/. The data supporting the findings of this study are available from the corresponding author upon a reasonable request.

## Abbreviations

AS: Atherosclerosis
HT: Hashimoto’s thyroiditis
GO: Gene ontology
KEGG: Kyoto Encyclopedia of Genes and Genomes
GEO: Gene Expression Omnibus
PPI: Protein–protein interaction
DEGs: Differentially expressed genes
WGCNA: weighted gene coexpression network
GSEA: Gene Set Enrichment Analysis
scRNA-seq: single-cell RNA sequencing
NES: normalized enrichment score
FDR: false positive rate
TOM: topological overlap matrix
MAD: median absolute deviation
ROC: Receiver Operating Characteristic
AUC: Area Under the Curve
t-SNE: t-distributed stochastic neighbor embedding
