# Supplementary material for: Exploring the comorbidity mechanisms between atherosclerosis and hashimoto’s thyroiditis based on microarray and single-cell sequencing analysis
Source: Sci Rep. 2025 Jan 13;15:1792. doi: 10.1038/s41598-025-85112-0 (PMC11730997; doi:10.1038/s41598-025-85112-0)
Supplement: Supplementary file 1 — Supplementary Information 1. [file 41598_2025_85112_MOESM1_ESM.pdf]

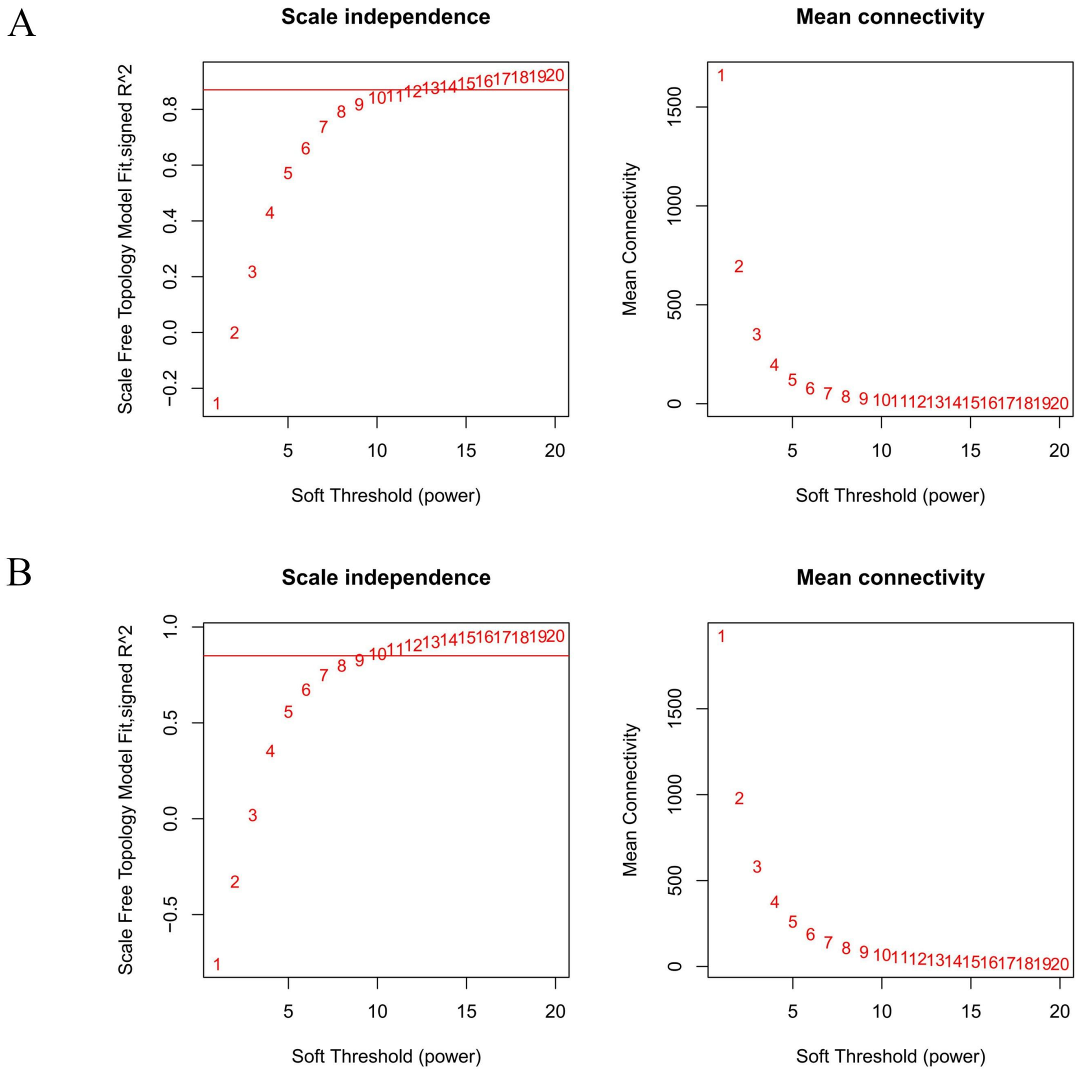

Supplementary File 1: (A) The determination of soft thresholding power in GSE28829. (B) The determination of soft thresholding power in GSE138198.
